# Timing of acute carotid artery stenting for tandem lesions in patients with acute ischemic stroke: A Maastricht Stroke Quality Registry (MaSQ-Registry) study

**DOI:** 10.1177/15910199241245166

**Published:** 2024-04-09

**Authors:** Sorina R. Simon, Robrecht R.M.M. Knapen, Martine T.B. Truijman, Robert J. van Oostenbrugge, Bart A.J.M. Wagemans, Wim H. van Zwam, Christiaan van der Leij

**Affiliations:** 1 Department of Radiology and Nuclear Medicine, 199236 Maastricht University Medical Center, Maastricht, The Netherlands; 2 118066 School for Cardiovascular Diseases (CARIM), Maastricht University, Maastricht, The Netherlands; 3 Department of Neurology, 199236 Maastricht University Medical Center, Maastricht, The Netherlands

**Keywords:** Carotid artery stenting, tandem lesions, ischemic stroke, endovascular treatment, intracranial thrombectomy

## Abstract

**Background:**

To better understand the influence of treatment strategies on outcomes for patients with tandem lesions undergoing acute internal carotid artery (ICA) stenting during endovascular treatment (EVT), this study compared clinical, technical, and safety outcomes in patients with acute ischemic stroke due to a large vessel occlusion (LVO) who underwent ICA stenting before versus after intracranial thrombectomy.

**Methods:**

This single-center retrospective cohort study included patients who underwent EVT due to a LVO and periprocedural ICA stenting for significant ICA stenosis or occlusion between September 2020 and January 2023. Data were extracted from the Maastricht Stroke Quality Registry (MaSQ-Registry). Primary outcome was the modified Rankin Scale (mRS) at 3 months. Secondary outcomes included procedure times, number of total thrombectomy attempts, first-attempt excellent recanalization rates (extended Thrombolysis In Cerebral Infarction (eTICI) ≥ 2C after one thrombectomy attempt), and safety outcomes.

**Results:**

This study included 50 patients. Thirty-one patients (62%) underwent ICA stenting before intracranial thrombectomy. No significant differences between both groups were found regarding mRS, total procedure time, number of total thrombectomy attempts, first-attempt excellent recanalization, or complications. Time between groin puncture and recanalization (reperfusion time) was significantly longer in patients who had ICA stenting before intracranial thrombectomy versus after intracranial thrombectomy (45 min versus 28 min, P = 0.004).

**Conclusion:**

ICA stenting after intracranial thrombectomy in patients with tandem lesions undergoing EVT did not lead to better patient outcomes compared to stenting before intracranial thrombectomy, despite shorter reperfusion times.

## Introduction

Patients with acute ischemic stroke (AIS) due to large vessel occlusion (LVO) undergoing endovascular treatment (EVT) may present with a tandem lesion, i.e. a significant stenosis or occlusion of the ipsilateral cervical internal carotid artery (ICA) next to the intracranial LVO, in up to 10–15%.^1^ Literature has shown that patients with tandem lesions have worse clinical outcome compared to patients with isolated LVO.^2^ Despite the high prevalence of tandem lesions in patients undergoing EVT, the optimal treatment strategy remains unclear.^3^ There is no consensus whether tandem lesions should be treated in the acute setting with stent placement, or in the delayed setting, i.e. deferred treatment, either with stenting or carotid endarterectomy.^4^ If stent placement is performed during EVT, there is no consensus whether the intracranial LVO or cervical carotid lesion should be treated first.^5^ Intracranial first approach may reduce time to recanalization and limit the size of the cerebral infarction, yet forms a risk of distal embolization.^4,5^ Extracranial first approach, during which stenting is performed in the ICA prior to thrombectomy, may improve flow-related recanalization of the intracranial lesion due to collateral flow.^5^

To better understand the influence of treatment strategies on outcomes for patients with tandem lesions undergoing acute ICA stenting during EVT, this study compared clinical, technical, and safety outcomes in patients with AIS due to LVO who underwent ICA stenting before versus after intracranial thrombectomy.

## Materials and methods

### Study design and patient selection

This single-center retrospective cohort study of prospectively registered data included all consecutive patients who underwent EVT due to a LVO of the anterior intracranial circulation in a tertiary academic hospital between September 2020 and January 2023. For quality purposes, data are registered in the Maastricht Stroke Quality Registry (MaSQ-Registry). Inclusion criteria for the current study were age ≥ 18 years; a significant stenosis (i.e. a stenosis of 50–99%) or occlusion of the ipsilateral extracranial ICA in combination with an anterior circulation occlusion of intracranial carotid artery (internal carotid artery terminus [ICA-T]) or M1 or M2 segment of middle cerebral artery (MCA) demonstrated by baseline computed tomographic (CT) angiography; groin puncture within 24 h after symptom onset; and periprocedural extracranial ICA stenting. During EVT, patients were treated with direct aspiration, stent retriever thrombectomy, or a combination of both techniques. Patients were included when at least a two-directional view on final digital subtraction angiography was available. Patients were excluded if only percutaneous transluminal angioplasty (PTA) was performed. An independent core lab consisting of two radiologists with more than 10 years of experience assessed all the imaging separately and scored the Alberta Stroke Program Early CT Score (ASPECTS) and collaterals,^6,7^ while they were blinded for all clinical findings and findings on the post-EVT (dual-energy) CT. The ASPECTS determines MCA stroke severity using CT data.^8^ The collaterals were graded from 0 to 3 (0: absent collateral supply to the occluded MCA territory; 1: collateral supply filling <50% but >0% of the occluded MCA territory; 2: collateral supply filling >50% but <100% of the occluded MCA territory; 3: 100% collateral supply of the occluded MCA territory). The study protocol was approved by the local medical ethics committee. The local medical ethics committee waived the need for obtaining informed consent.

### Treatment

All patients underwent periprocedural ICA stenting. PTA prior to or after stenting was performed whenever necessary. In some patients, a balloon guide catheter was used to enable flow arrest during clot removal or during stent placement to prevent distal emboli. The antiplatelet regimen in patients with ICA stenting included 500 mg acetylsalicylic acid (ASA) IV during the procedure and 300 mg clopidogrel loading after the post-EVT CT. Subsequently patients received 3 months of dual antiplatelet therapy (100 mg ASA and 75 mg clopidogrel) and lifelong monotherapy with 75 mg clopidogrel.

### Outcome measures

The primary outcome measure was the modified Rankin Scale (mRS) score at 3 months follow-up.^9^ The mRS ranges from 0 (no disability) to 6 (death). The mRS was scored as a quality measurement (standard care) approximately 90 days after EVT by a trained (research) nurse. Secondary outcome measures included procedure times, number of total thrombectomy attempts, first-attempt excellent recanalization rates, and safety outcomes. Reperfusion time was defined as time between groin puncture and recanalization. Total procedure time was defined as time between groin puncture and end of the procedure (vascular closure). Recanalization was assessed using the extended Thrombolysis In Cerebral Infarction (eTICI) grade, a scale ranging from no reperfusion (eTICI 0) to complete reperfusion (eTICI 3).^10^ Excellent recanalization was defined by eTICI grade 2C (90–99% reperfusion) or 3 (complete reperfusion). First-attempt excellent recanalization was defined by eTICI grade 2C or 3 after one thrombectomy attempt. Safety outcomes were the occurrence of periprocedural complications and intracranial hemorrhage on the post-EVT CT. Periprocedural complications were defined as adverse events resulting from the endovascular procedures such as the occurrence of distal emboli in non-involved arteries, perforations, and dissections.

### Statistical analysis

Baseline characteristics were analyzed by using standard statistics. Baseline characteristics were reported as frequencies and percentages for categorical variables, and as means and standard deviation (SD) or medians and interquartile ranges (IQRs) for continuous variables. Where appropriate, missing values were reported. Univariate analysis explored the baseline differences between patients who underwent ICA stenting before versus after intracranial thrombectomy, using independent samples t-test or Mann–Whitney U test for continuous variables, and Chi-square or Fisher's exact test for categorical variables. Regarding the primary outcome, ordinal regression analysis was performed to compare the effect of stenting before versus after intracranial thrombectomy on the mRS score at 3 months. A two-sided *P*-value ≤ 0.05 was considered statistically significant. Statistical analyses were performed using IBM SPSS Statistics for Windows, version 25 (IBM, Armonk, NY).

## Results

### Patient population

**Figure 1.**
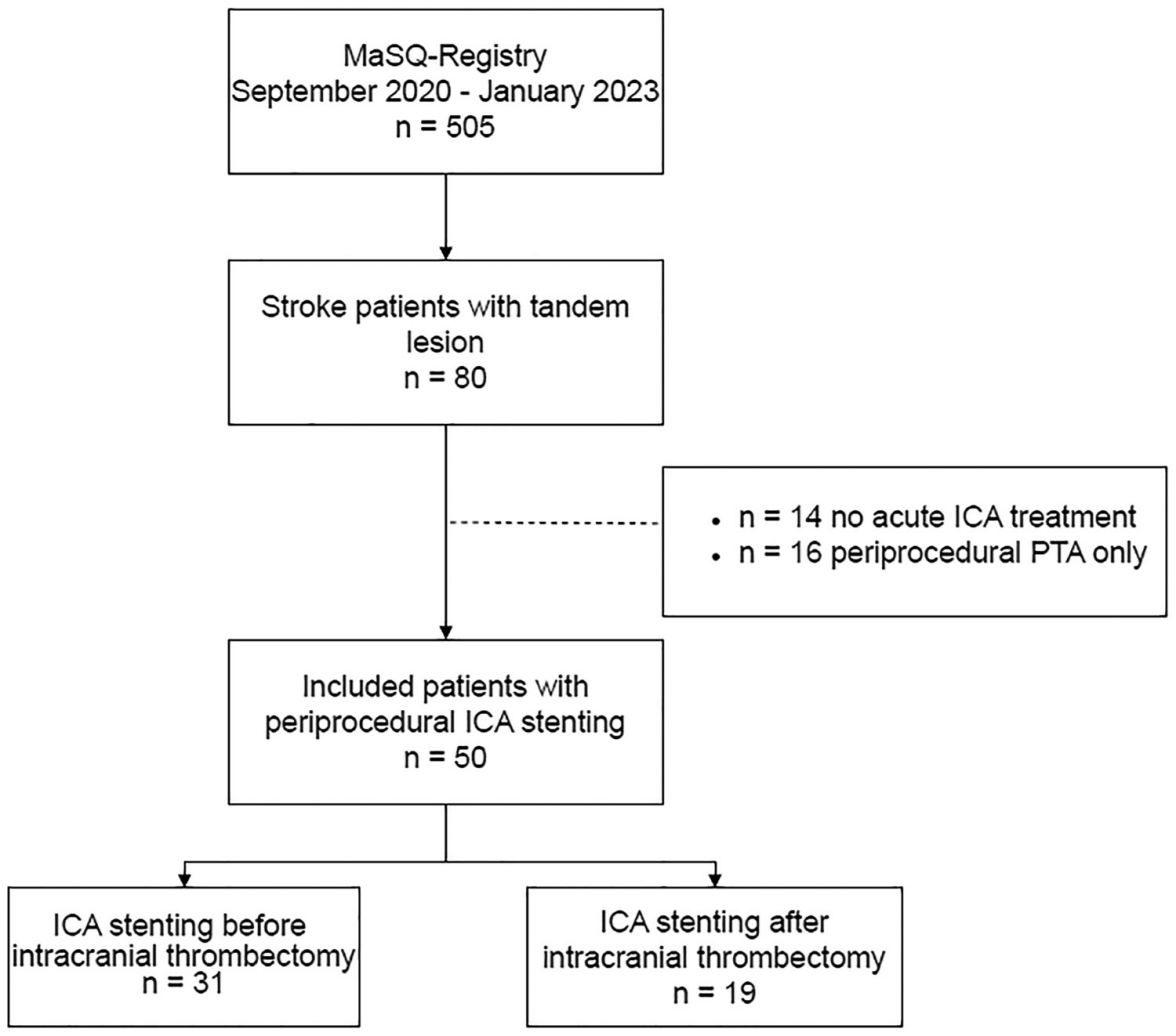
Flowchart of included patients. ICA: internal carotid artery; EVT: endovascular treatment.

In the MaSQ-Registry, which included 505 patients who underwent EVT up to January 2023, 80 patients (15.8%) had a tandem lesion during EVT. Within this group, 14 patients (17.5%) did not receive acute carotid artery treatment during EVT, 16 patients (20%) were treated with PTA only, and 50 patients (62.5%) were treated with acute carotid stenting (Figure 1). These 50 patients with periprocedural ICA stenting were analyzed in this study. Patient characteristics are presented in Table 1, including univariate analysis. Median age of the patients was 73 years (IQR, 68–79 years) and the majority was male (n = 33; 66%). Forty patients (80%) were referred from other hospitals. Five patients (10%) presented between 6 and 24 h after symptom onset or last seen well. The median baseline National Institutes of Health Stroke Scale (NIHSS) score was 13 (IQR, 7–18). On baseline CTA, the majority of patients presented with an occlusion of the M1 segment (n = 26) (52%). Twenty-six patients (52%) had an ipsilateral ICA significant stenosis, whereas 24 patients (48%) presented with an ipsilateral ICA occlusion. Thirty-one patients (62%) underwent ICA stenting before intracranial thrombectomy, and 19 patients (38%) underwent ICA stenting after intracranial thrombectomy. A balloon guide catheter was used significantly more frequently in patients who had ICA stenting after intracranial thrombectomy versus before intracranial thrombectomy (84.2% versus 35.5%. *P *= 0.001). The distribution of the eight treating physicians is presented in supplemental material (Table S1). No statistically significant difference was observed regarding extracranial or intracranial first approach among the physicians involved (*P *= 0.33).

**Table 1.** Characteristics of patients who underwent periprocedural ICA stenting before (n = 31) versus after (n = 19) intracranial thrombectomy, including univariate analysis.

Variable	ICA stenting before intracranial thrombectomy (n = 31)	ICA stenting after intracranial thrombectomy (n = 19)	*P-*value
**Median age, years**	72 (68–79)	75 (62–81)	0.84
**Male sex**	18 (58.1%)	15 (78.9%)	0.13
**Hypertension**	16 (51.6%)	6 (31.6%)	0.17
**Atrial fibrillation** ^a^	1 (3.4%)	1 (5.3%)	1.00
**Previous stroke** ^b^	2 (6.7%)	4 (21.1%)	0.11
**Pre-stroke mRS score** ^c^			
Median (IQR)	0 (0–1)	0 (0–1)	0.99
Ordinal			0.53
0	11 (68.8%)	10 (62.5%)	
1	2 (12.5%)	5 (31.3%)	
≥2	3 (18.7%)	1 (6.2%)	
**Median baseline NIHSS score**	11 (6–17)	15 (8–20)	0.19
**Intravenous alteplase treatment**	19 (61.3%)	13 (68.4%)	0.61
**Collaterals** ^d^			0.11
Grade 0	0 (0%)	0 (0%)	
Grade 1	11 (37.9%)	12 (63.2%)	
Grade 2	16 (55.2%)	6 (31.6%)	
Grade 3	2 (6.9%)	1 (5.2%)	
**Median ASPECTS**	8 (6–9)	8 (6–10)	0.79
**Location of stroke in left hemisphere**	14 (45.2%)	9 (47.4%)	0.88
**Location of intracranial occlusion on baseline CT**			0.37
ICA-T	3 (9.7%)	5 (26.3%)	
ICA-T with involvement of M1 segment	2 (6.5%)	1 (5.3%)	
MCA M1 segment	16 (51.6%)	10 (52.6%)	
MCA M2 segment	10 (32.3%)	3 (15.8%)	
**Extracranial ICA occlusion on baseline CT**	14 (45.2%)	10 (52.6%)	0.61
**Extracranial significant ICA stenosis on baseline CT**	17 (54.8%)	9 (47.4%)	0.61
**Median time from onset to groin puncture, minutes**	195 (153–310)	180 (130–503)	0.72
**First-line thrombectomy technique** ^e^			0.52
Direct aspiration	8 (29.6%)	7 (38.9%)	
Stent retriever	0 (0%)	0 (0%)	
Combination of direct aspiration and stent retriever	19 (70.4%)	11 (61.1%)	
**Balloon guide catheter**	11 (35.5%)	16 (84.2%)	**0**.**001**
**ICA stent type**			0.67
RX Acculink™ Carotid Stent System (Abbott Vascular, Santa Clara, US)	14 (45.2%)	10 (52.6%)	
Roadsaver™ Carotid Artery Stent (Terumo, Tokyo, Japan)	17 (54.8%)	9 (47.4%)	

Note: Data are reported as n (%), mean (SD) or median (interquartile range) unless otherwise stated. Bold value indicates the statistically significant of *P* < 0.05

ICA: internal carotid artery; IQR: interquartile range; mRS: modified Rankin Scale; NIHSS: National Institutes of Health Stroke Scale; ASPECTS: Alberta Stroke Program Early Computed Tomography score; ICA-T: terminus of internal carotid artery; MCA: middle cerebral artery; CT: computed tomographic.

a Data were missing for two patients.

b Data were missing for one patient.

c Data were missing for 18 patients.

d Data were missing for two patients.

e Data were missing for five patients.

### Clinical outcomes

**Figure 2.**
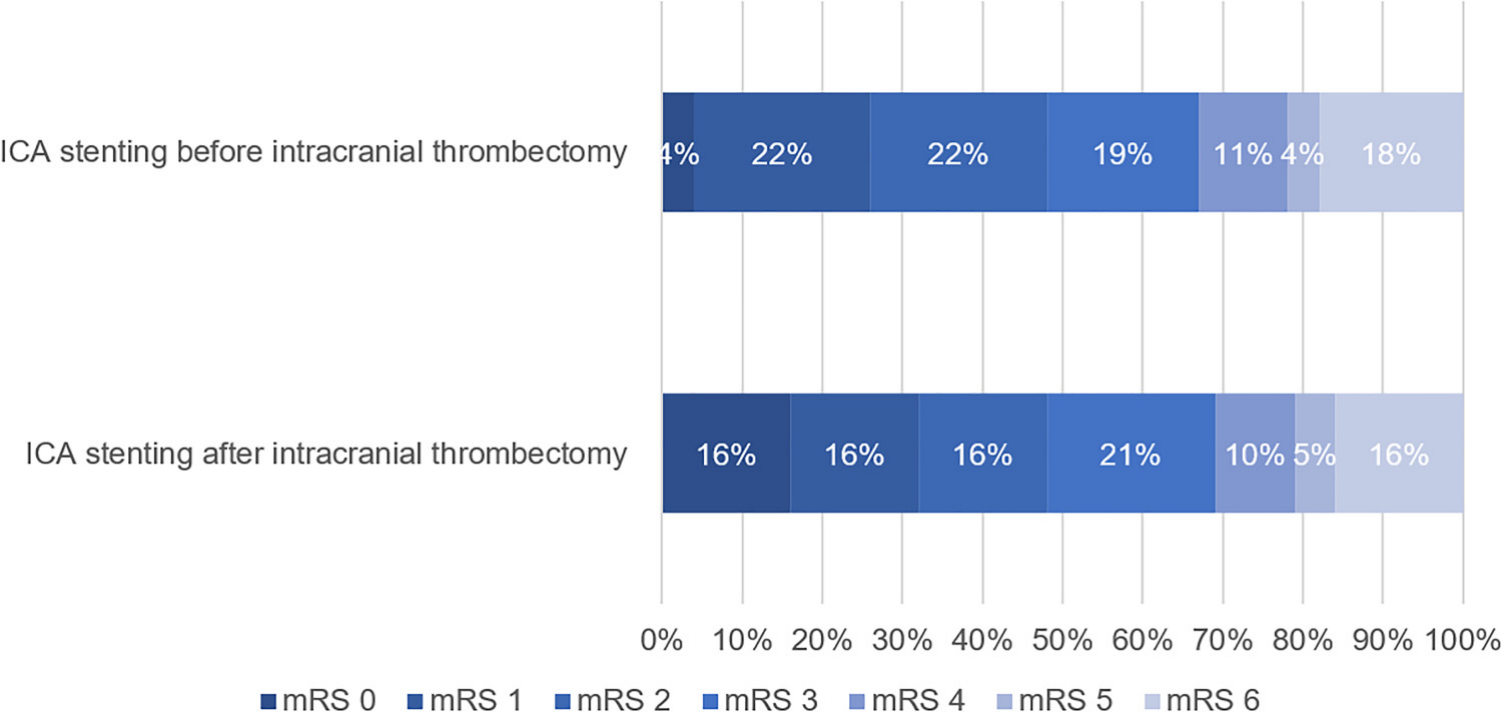
Distribution of modified Rankin Scale (mRS) at 3 months between patients with periprocedural ICA stenting before (n = 31) versus after (n = 19) intracranial thrombectomy^a^. ICA: internal carotid artery; mRS: modified Rankin Scale. Ordinal logistic regression showed no significant difference in ordinal mRS score after 90 days (common odds ratio, 0.81 [95% CI 0.29–2.30]). ^a^ mRS score was missing for four patients.

No significant differences between both groups were found regarding the median mRS score at 3 months (*P *= 0.70) (Table 2). The distribution of the mRS between the groups is presented in Figure 2. Ordinal logistic regression showed no significant difference in ordinal mRS score after 90 days (common odds ratio, 0.81 [95% CI 0.29–2.30], *P *= 0.69). Thirteen patients (48.1%) who underwent ICA stenting before intracranial thrombectomy had a favorable clinical outcome (mRS 0–2), whereas nine patients (47.4%) who underwent ICA stenting after intracranial thrombectomy had a favorable clinical outcome.

**Table 2.** Primary and secondary outcomes for patients who underwent periprocedural ICA stenting before (n = 31) versus after (n = 19) intracranial thrombectomy, including univariate analysis.

Outcome	ICA stenting before intracranial thrombectomy (n = 31)	ICA stenting after intracranial thrombectomy (n = 19)	*P-*value
**mRS score at 3 months** ^a^			
Median (IQR)	3 (1–4)	3 (1–4)	0.70
Ordinal			0.69
0	1 (3.7%)	3 (15.8%)	
1	6 (22.2%)	3 (15.8%)	
2	6 (22.2%)	3 (15.8%)	
3	5 (18.5%)	4 (21%)	
4	3 (11.2%)	2 (10.5%)	
5	1 (3.7%)	1 (5.3%)	
6	5 (18.5%)	3 (15.8%)	
**Median reperfusion time (time between groin puncture and recanalization), minutes**	45 (35–63)	28 (20–53)	**0**.**004**
**Median total procedure time (time between groin puncture and end of procedure), minutes**	52 (40–69)	58 (46–73)	0.18
**Median number of total thrombectomy attempts**	1 (1–3)	2 (1–3)	0.37
**Post-EVT eTICI**			0.38
Grade 0	0 (0%)	1 (5.3%)	
Grade 1	0 (0%)	0 (0%)	
Grade 2A	2 (6.5%)	1 (5.3%)	
Grade 2B	9 (29%)	1 (5.3%)	
Grade 2C	15 (48.4%)	13 (68.4%)	
Grade 3	5 (16.1%)	3 (15.7%)	
Excellent recanalization (grade 2C or 3)	20 (64.5%)	16 (84.1%	
**First-attempt excellent recanalization (eTICI grade 2C or 3 after one thrombectomy attempt)**	8 (25.8%)	5 (26.3%)	0.97
**Periprocedural complications**	8 (25.8%)	6 (31.6%)	0.66
Distal embolization	3 (9.7%)	4 (21.1%)	0.29
**Intracranial hemorrhage on post-EVT CT**	5 (16.1%)	5 (26.3%)	0.39

Note: Data are reported as n (%) or median (interquartile range) unless otherwise stated. Bold value indicates the statistically significant of *P* < 0.05.

ICA: internal carotid artery; mRS: modified Rankin Scale; IQR: interquartile range; EVT: endovascular treatment; eTICI: extended Thrombolysis In Cerebral Infarction grade; CT: computed tomographic.

a mRS score was missing for four patients.

### Technical and safety outcomes

No significant differences between both groups were found regarding number of total thrombectomy attempts, first-attempt excellent recanalization rates, periprocedural complications, or intracranial hemorrhage on the post-EVT CT (Table 2). Time between groin puncture and recanalization (reperfusion time) was significantly longer in patients who had ICA stenting before intracranial thrombectomy versus after intracranial thrombectomy (median of 45 min versus 28 min, *P *= 0.004). Despite this difference in reperfusion time, no statistical difference was found regarding total procedure time between both groups (*P *= 0.18). While distal embolization occurred twice as frequent in patients with ICA stenting after intracranial thrombectomy compared to patients with ICA stenting before intracranial thrombectomy (21.1% versus 9.7%), no statistically significant difference was observed in distal embolization rates (*P *= 0.29).

## Discussion

The optimal management of tandem lesions in patients with a LVO undergoing EVT remains unclear. In this post hoc analysis of an EVT registry, we compared clinical, technical, and safety outcomes in patients with tandem lesions who underwent ICA stenting before versus after intracranial thrombectomy.

No significant differences were found regarding clinical outcome at 3 months and safety outcomes. When looking at technical outcomes, patients who underwent ICA stenting before intracranial thrombectomy had significantly longer reperfusion times compared to patients who underwent ICA stenting after intracranial thrombectomy. This finding may be explained by the duration of stent placement prior to intracranial thrombectomy. However, no significant difference was observed in total procedure time. There was a trend for less hemorragic transformation and less passes in the antegrade group. A balloon guide catheter was used significantly more in patients who had ICA stenting after intracranial thrombectomy, and a trend was observed towards more frequent distal embolization in this subgroup compared to patients with ICA stenting before intracranial thrombectomy. This is an interesting finding as the use of a balloon guide catheter is expected to reduce the risk of distal embolization.^11^ This observation might also suggest that distal embolization is a relevant problem with the retrograde approach. This difference in distal embolization rates might be even higher if balloon guide catheters had been used equally often in both groups, as it is hypothesized that the outcome using an antegrade approach could have been better if the rates of balloon guide catheter use had been similar.

Few studies directly compared extracranial first versus intracranial first approach in patients undergoing periprocedural ICA stenting during EVT. Some studies reported findings in favor of an intracranial first approach such as shorter reperfusion time,^12^ shorter angiography time,^12^ higher recanalization rate,^13,14^ and better functional outcome.^14^ A study by Marko et al., however, reported longer total procedure time in patients with an intracranial first approach.^15^ On the contrary, other studies did not find differences between both groups regarding reperfusion time,^16^ recanalization rate,^17^ or functional outcome.^18^ While an increasing number of studies have compared intracranial first versus extracranial first approach, studies often fail to distinguish between stenting and PTA only as treatment options,^5,19^ impeding proper comparison with our study.

Advantages of an intracranial first approach include shorter reperfusion time, thus limiting cerebral infarction size.^4^ An important disadvantage of intracranial first approach concerns the risk of dislodgment of a thrombus and distal embolization during subsequent ICA stent deployment. However, in patients with an intracranial occlusion located in the ICA-T, there is no risk of distal embolization, as emboli due to ICA stenting become trapped in the pre-existing ICA-T occlusion. Thus, extracranial first approach may be favored in patients with an ICA-T occlusion. In this study, still the majority of patients with ICA-T occlusions were treated with a retrograde approach. We do not have a clear explanation for this. Preference of the treating physician might have played a role. One other explanation might be that antegrade recanalization already changed the position of the clot, by pushing it forward, hereby lowering the number of ICA-T occlusions. This might also explain the higher proportion of M2 occlusions in the antegrade group. It is known that stenting/re-establishing carotid flow has impact on intracranial occlusions: there are reported cases of MCA occlusions that vanish after stenting.^20,21^

Proponents of the extracranial first approach argue in favor of improved flow-related recanalization of the intracranial lesion.^5,17,20^ Spontaneous intracranial recanalization immediately after ICA stenting, thus eliminating the need for intracranial intervention, has been previously described in literature.^20,21^ Likewise, we observed spontaneous intracranial recanalization in three patients in our study, which might be explained by increased distal perfusion through collaterals. Another advantage of the extracranial first approach is improved access for the treatment of the intracranial lesion due to easier passage across the ICA,^17,20^ although some studies reported on stent retrievers getting caught in the ICA stent which may disrupt the thrombectomy device.^12,22^

The present study is valuable as it adds to the growing body of evidence on antegrade versus retrograde approach in acute carotid artery stenting during EVT. While many previous articles freely combine PTA only and stenting, our paper focusses exclusively on acute ICA stenting. In contrast to other studies, our paper also includes patients who presented in the late window (i.e. between 6 and 24 h after symptom onset or last seen well) which is an important subgroup, as late window EVTs have increased substantially over time.

Our study has some limitations. The study design concerned a retrospective observational cohort study of prospectively registered data. We investigated patients who underwent periprocedural extracranial ICA stenting due to a significant stenosis or occlusion. No distinction was made between the different types of tandem lesions (i.e. a significant stenosis versus occlusion of the ICA), though no statistical difference was found regarding baseline characteristics. In addition, the rationale of the treatment strategy, i.e. extracranial first versus intracranial first approach, is unknown. The choice of treatment strategy was based on preference of the treating physician, as was the use of a balloon guide catheter. Each treating physician also selected the preferred materials for ICA stenting including stent type, length of stent, and whether PTA was performed additionally. However, the MaSQ-Registry represents daily clinical practice in acute endovascular stroke treatment. Future studies should further explore the optimal treatment strategy in this patient population. We hope to investigate the potential effect of these variables in a larger sample size in the future.

## Conclusion

In patients with tandem lesions undergoing EVT with periprocedural ICA stenting, ICA stenting after thrombectomy did not lead to better patient outcomes, despite shorter reperfusion times. No significant differences were found regarding clinical or safety outcomes. Future studies should further explore the optimal treatment strategy in this patient population.

## Supplemental Material

sj-docx-1-ine-10.1177_15910199241245166 - Supplemental material for Timing of acute carotid artery stenting for tandem lesions in patients with acute ischemic stroke: A Maastricht Stroke Quality Registry (MaSQ-Registry) study Supplemental material, sj-docx-1-ine-10.1177_15910199241245166 for Timing of acute carotid artery stenting for tandem lesions in patients with acute ischemic stroke: A Maastricht Stroke Quality Registry (MaSQ-Registry) study by Sorina R. Simon, Robrecht R.M.M. Knapen, Martine T.B. Truijman, Robert J. van Oostenbrugge, Bart A.J.M. Wagemans, Wim H. van Zwam and Christiaan van der Leij in Interventional Neuroradiology
