# Supplementary material for: Timing of acute carotid artery stenting for tandem lesions in patients with acute ischemic stroke: A Maastricht Stroke Quality Registry (MaSQ-Registry) study
Source: Interv Neuroradiol. 2024 Apr 9:15910199241245166. Online ahead of print. doi: 10.1177/15910199241245166 (PMC11571490; doi:10.1177/15910199241245166)
Supplement: sj-docx-1-ine-10.1177_15910199241245166 - Supplemental material for Timing of acute carotid artery stenting for tandem lesions in patients with acute ischemic stroke: A Maastricht Stroke Quality Registry (MaSQ-Registry) study [file sj-docx-1-ine-10.1177_15910199241245166.docx]

**Table S1.** Distribution of physicians who performed periprocedural ICA stenting before (n = 31) versus after (n = 19) intracranial thrombectomy

| **Physician** | **ICA stenting before intracranial thrombectomy (n = 31)** | **ICA stenting after intracranial thrombectomy (n = 19)** |
| --- | --- | --- |
| 1 | 4 | 1 |
| 2 | 5 | 2 |
| 3 | 0 | 2 |
| 4 | 7 | 2 |
| 5 | 2 | 2 |
| 6 | 4 | 6 |
| 7 | 5 | 1 |
| 8 | 4 | 3 |

ICA = internal carotid artery
